# Low uptake of COVID-19 booster doses among elderly cancer patients in China: A multicentre cross-sectional study

**DOI:** 10.7189/jogh.14.05010

**Published:** 2024-02-02

**Authors:** Ruiyu Chai, Jianzhou Yang, Rila Su, Xinquan Lan, Moxin Song, Lei Zhang, Junjie Xu

**Affiliations:** 1 Clinical Research Academy, Peking University Shenzhen Hospital, Shenzhen, Guangdong Province, China; 2 Department of Epidemiology and Biostatistics, School of Public Health, Jilin University, Changchun, Jilin Province, China; 3 Department of Public Health and Preventive Medicine, Changzhi Medical College, Changzhi, Shanxi Province, China; 4 Department of Oncology, Peking University Shenzhen Hospital, Shenzhen, Guangdong Province, China; 5 Department of Epidemiology, China Medical University, Shenyang, Liaoning Province, China; 6 Cancer Center of Inner Mongolia People's Hospital, Hohhot, Inner Mongolia, China; 7 John Hopkins Bloomberg School of Public Health, Baltimore, Maryland, USA

## Abstract

**Background:**

Vaccination is a crucial measure to control the spread of coronavirus disease 2019 (COVID-19) pandemic. The elderly and cancer populations both are more susceptible to SARS-CoV-2 and have higher mortality. However, the uptake of COVID-19 vaccine booster doses among elderly cancer patients remains unclear. This study aimed to investigate the prevalence and associates of COVID-19 vaccine booster doses uptake in elderly cancer patients.

**Methods:**

A multi-center cross-sectional survey was conducted in four general populations of China province from April to June 2022. Demographic and clinical characteristics, as well as COVID-19 vaccination status and reasons for not uptake booster doses, were collected through face-to-face interviews and medical records. Multivariable logistic regression models were performed to explore the associates of the first COVID-19 booster dose vaccination uptake of cancer patients.

**Results:**

A total of 893 cancer patients were eventually included in this study, of which 279 (31.24%) were aged 65 or older and 614 (68.76%) were under 65 years old. The proportion of the first COVID-19 vaccine booster dose among cancer patients aged 65 and above was lower than among adults aged 65 (23.66 vs. 31.92%). Factors affecting individual-level variables among the aged 65 and above cancer patients group whether to uptake the first COVID-19 booster dose were negative attitudes toward COVID-19 vaccine booster dose, perceived subjective norm, perceived behavioural control, and other types of chronic disease. There is no significant difference in the incidence of related adverse reactions between the two age groups (*P* = 0.19).

**Conclusions:**

Low uptake of COVID-19 vaccine booster doses among elderly cancer patients is a significant concern and implies high susceptibility and high fatality when facing the emergence of SARS Cov-2 outbreak. Efforts to improve vaccine education and accessibility, particularly in rural areas, may help increase uptake and reduce the spread of SARS-Cov-2.

Coronavirus disease 2019 (COVID-19) has caused over 762 million confirmed cases and nearly seven million deaths worldwide, as the World Health Organization (WHO) reported on 5 April 2023 [1]. This has led to widespread health, economic, and social insecurity globally. Research has shown that older patients and/or those with comorbidities, especially cancer, are at substantially higher mortality and more severe COVID-19 clinical outcomes [2,3]. More than 81.0 and 90.1% of COVID-19 deaths in the USA and China occurred in individuals over 65 years, respectively [4,5]. Additionally, studies have demonstrated that cancer patients are more vulnerable to COVID-19 infection and have a 1.8 times higher mortality rate than non-cancer patients [6–9]. Therefore, vaccination is crucial for elderly cancer patients to prevent infection.

Vaccination is a vital strategy against COVID-19, triggering a protective immunological response and reducing the risk of severe COVID-19 clinical events [10,11]. A US cohort study with an average age of 74.1 years found that cancer patients who uptake a second dose of the COVID-19 vaccine had a 58% lower risk of infection than those who did not [12]. Real-world evidence has also demonstrated that completing the COVID-19 vaccination markedly reduces the risk of breakthrough infection in cancer patients [13]. Nevertheless, while COVID-19 vaccines have been shown to reduce mortality rates, relevant research has indicated that anti-SARS-CoV-2 antibody levels decline significantly after a few months, even though patients who are older and/or have cancer (including non-cancer individuals) uptake second primary doses [14,15]. Therefore, COVID-19 booster doses (i.e. booster vaccine doses after primary vaccination) are needed to augment vaccine efficacy and reduce infection risk, particularly in those at higher risk of severe disease.

Recently, most authoritative institutions issued vaccination guidelines and recommended that older patients and cancer should uptake COVID-19 vaccine booster doses, including the US Centers for Disease Control and Prevention (CDC) [16], the National Comprehensive Cancer Network (NCCN) [17], European Society of Medical Oncology (ESMO) [18]. Moreover, in November 2022, China also updated its COVID-19 vaccination guideline, recommending booster doses for older people [19]. For cancer patients, a safe and effective COVID-19 vaccine and high vaccination coverage are essential. However, some cancer patients hold doubts and negative views on vaccination. A longitudinal survey among US cancer patients revealed that they were concerned about vaccine effectiveness, safety/side effects, and distrust of COVID-19 vaccines [20]. Similarly, a study in the Middle East and North Africa found that individuals who refused the booster doses were uncertain about its safety and believed it was unnecessary, immunosuppressive, and had side effects from previous COVID-19 vaccine doses [21]. Ironically, immunosuppressed patients are more likely to refuse COVID-19 booster doses compared to healthy individuals despite their greater need for the COVID-19 vaccine. Additionally, a survey in Hong Kong indicated that older age and the presence of chronic conditions were associated with poorer responses to the first COVID-19 vaccine booster doses [22]. In contrast, individuals who perceived government promotional materials as helpful in addressing their concerns had higher uptake of the COVID-19 vaccine booster dose and made more informed decisions.

China, with the world's largest elderly population and a high incidence of cancer, urgently needs to understand the acceptance of the first COVID-19 booster dose among these vulnerable groups [23]. However, to our knowledge, no studies or reports on this topic have yet been conducted in China. This presents a significant research gap.

To address this gap, we propose a study using the Health Belief Model (HBM) as a theoretical framework. The objective of our study is to evaluate the attitudes of individuals aged 65 and above with cancer in China towards the uptake of the first COVID-19 booster dose and to identify the relevant factors influencing their decisions. This is of great guiding significance to answer the internal reasons for the strange phenomenon that the population has a high risk of COVID-19 infection and death, but a very low COVID-19 vaccination rate. The findings will provide a foundation for developing COVID-19 vaccination policies specifically targeted at elderly cancer patients, a group that is of vital importance in the fight against the pandemic.

## METHODS

### Study design

This cross-sectional study was conducted across multiple centers. This study used a multi-stage convenience sampling method. First, we select four major cities inconvenience. Subsequently, we selected one comprehensive hospital with the highest number of cancer patients in the four selected cities (these tertiary hospitals included two in North China (Shanxi: Heping Hospital Affiliated to Changzhi Medical College; Inner Mongolia: Inner Mongolia: Inner Mongolia People's Hospital), one in Northwest China (Xinjiang: The First Affiliated Hospital of Xinjiang Medical University), and one in South China (Guangdong: Peking University Shenzhen Hospital)). All hospitalised patients in the oncology department of the selected hospital during the study period were the potential participants of this study. Face-to-face interviews were conducted to collect questionnaire data. Recruitment took place between April and June 2022 at four large tertiary hospitals in three regions of China.

### Participants

The study enrolled individuals aged 18 years and above who had been diagnosed with cancer and were residing in one of the four participating hospitals. Eligible participants voluntarily participated in the survey and provided written informed consent. Exclusion criteria included: 1) patients with lymphoma, leukemia during acute attacks, or chemotherapy and 2) those who are unable to communicate effectively with researchers.

### Recruitment and data collection

In the participating hospitals, the medical staff actively contacted all hospitalised patients diagnosed with cancer in the department of oncology. After determining whether these patients were suitable to participate in the study, the medical staff explained the purpose and process of the study to them and invited them to face-to-face interviews. Patients were told that the information they provided would be confidential and that they could opt out of the study at any time. Choosing not to participate will not affect their normal medical services. Finally, patients were asked to sign written informed consent, indicating that they understood the study and voluntarily participated. To ensure the unified standard and quality of the study, we conducted a project training for all the staff responsible for questionnaire survey and other data collection at the beginning of this study.

### Instrumentation development of the questionnaire

To guarantee the accuracy and professionalism of the questionnaire, an expert team comprising two public health specialists and an epidemiologist focusing on cancer formulated and assessed all the questions. The questionnaire included the following sections: (1) background characteristics (including socio-demographic characteristics, cancer-related characteristics, presence of other chronic diseases, Yes vs. No, etc.); (2) uptake of the COVID-19 vaccine booster dose (the third dose); (3) hesitancy to receive COVID-19 vaccine booster doses; (4) individual-level variables (positive attitudes toward COVID-19 vaccine booster dose, negative attitudes toward COVID-19 vaccine booster, perceived subjective norm related to COVID-19 vaccine booster dose, perceived behavioural control to receive a COVID-19 vaccine booster dose); (5) vaccination fatigue; (6) frequency of thoughtful consideration about the veracity of COVID-19-specific information; (7) current treatment for cancer; (8) type of other chronic diseases; and (9) adverse reactions of uptake of the first COVID-19 vaccine booster dose. Individual-level variables section was based on the HBM. The HBM scale in both English language and Chinese language has been widely used to explore the public's vaccination beliefs and vaccination intentions, revealing important perceptual factors such as perceived susceptibility, perceived severity, perceived barriers, perceived benefits, self-efficacy, and cues to action [24,25]. Given the strong correlation between various factors of the original HBM, this study incorporates the following structures of the HBM: perceived severity, perceived benefits, self-efficacy, and cues to action. Moreover, each construct of (4) was assessed using a five-point Likert scale. Vaccine hesitancy was defined as the refusal, reluctance, or delay in acceptance of vaccinate despite vaccine availability [26]. Hesitancy is positioned between those who unquestionably accept all vaccines and those who outright refuse [27]. In this study, the first COVID-19 vaccine booster dose hesitancy was defined as ‘very unlikely’, ‘unlikely’, or ‘neutral’ to uptake the first COVID-19 vaccine booster dose [28,29].

### The sample size estimation

We calculated the sample size using a simple randomised sampling method formula. The ɑ was the significance level; if ɑ was taken to 0.05, Z_1-ɑ/2_ could be taken to 1.96; δ was the allowable error, δ was taken to 0.05. We assumed the estimated COVID-19 vaccine booster dose rate for cancer patients aged 65 and above was 50% [30]. Due to the convenience sampling method used in this survey, we subsequently multiplied the preliminary calculated sample size by a design effect (deff) coefficient. Based on a clustering sampling method, we used the deff to further calculate the sample size. The deff was defined as the ratio of the variance taking into account the clustering sample design and the variance of a simple random sample design with the same number of observations; the deff was taken to 1 based on the previous study [21]. Eventually, a sample size of 385 was initially generated based on a clustering sample study design. A minimum sample size of 534 was acquired after considering the no response rate of participants (20%) and the portion of unacceptable responses (10%). The sample size formula can be expressed as follows:



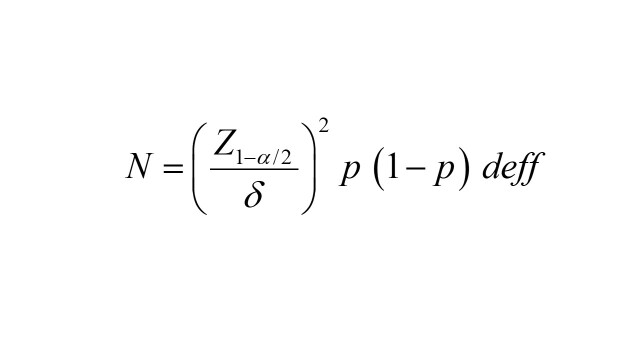



### Statistical analysis

We used the Kolmogorov-Smirnov test to check the normality of continuous variables. If the *P*-value of the Kolmogorov-Smirnov test is greater than 0.05, it is considered that the data conforms to a normal distribution, while if *P* < 0.05, it is considered that the data does not conform to a normal distribution. For normally distributed variables, we used mean and standard deviation; for others, we used median and interquartile range. The test results, with *P* > 0.05, suggest our data follows a normal distribution. Descriptive statistics, Chi square test (χ^2^), and Fisher precision probability test were utilised to summarise and determine the associations between the outcomes of interest and the different explanatory Univariate logistic regression was used to assess the associations between background characteristics and the uptake of the first COVID-19 vaccine booster dose in participants aged over 65 years and those aged <65, respectively. After adjusting for background characteristics with *P* < 0.05 in the univariate analysis, multivariable logistic regression was performed to compute the adjusted odds ratio (AOR) of factors for the first COVID-19 vaccine booster dose acceptance in the aged over 65 years and aged <65, respectively. All data analyses were performed using IBM SPSS software (version 24.0; IBM Corporation, Armonk, New York, USA). The significance level was set up at a two-tailed *P* < 0.05.

## RESULTS

### Background characteristics

During the study period, 1018 cancer patients were identified as eligible based on the inclusion and exclusion criteria. Of these patients, 125 refused participations in this study due to various reasons, including time constraints, personal issues, and lack of interest. Thus, our analysis ultimately included 893 cancer patients who successfully completed interviews, comprising 31.24% aged 65 and above, and 68.76% under 65 years old. The elderly group had a higher proportion of males, Han ethnicity, and individuals with an education level of junior high or below, compared to the younger group (63.08 vs. 50.49%, 86.38 vs. 81.76%, and 74.55 vs. 63.68%). Regarding cancer-related characteristics, the elderly group had a higher prevalence of other cancers and metastatic cancers than the younger group (35.84 vs. 42.67% and 92.51 vs. 26.06%, respectively). Furthermore, the elderly group had a higher burden of co-morbidities. Other characteristics had no significant differences between those aged 65 and above and those under 65 (*P* > 0.05) (**Table 1**).

**Table 1:** Background characteristics of multi-centre cancer participants (n = 893)

	> = 65 y (n = 279)	<65 y (n = 614)	OR (95% CI)	*P*-value
	**n (%)**	**n (%)**		
**Sociodemographic characteristics**				
Gender				
*Male*	176 (63.08)	310 (50.49)	1	
*Female*	103 (36.92)	304 (49.51)	1.90 (1.46–2.49)	<0.001*
Study site				
*Xinjiang*	91 (32.62)	190 (30.94)	1	
*Inner Mongolia*	73 (26.16)	134 (21.82)	0.88 (0.60–1.28)	0.51
*Shanxi*	79 (28.32)	126 (20.52)	0.76 (0.52–1.11)	0.16
*Guangdong*	36 (12.90)	164 (26.71)	2.18 (1.41–3.38)	<0.001*
Ethnicity				
*Other ethnic minorities*	38 (13.62)	112 (18.24)	1	
*Han ethnicity*	241 (86.38)	502 (81.76)	0.71 (0.47–1.05)	0.09
Education level				
*Junior high or below*	208 (74.55)	391 (63.68)	1	
*Senior high or equivalent*	49 (17.56)	117 (19.06)	1.27 (0.87–1.85)	0.21
*College and above*	22 (7.89)	106 (17.26)	2.56 (1.57–4.18)	<0.001*
Relationship status				
*Single/divorced/widowed*	28 (10.04)	49 (7.98)	1	
*Married*	251 (89.96)	565 (92.02)	1.29 (0.79–2.09)	0.31
Employment status				
*Full-time*	2 (0.72)	73 (11.89)	1	
*Part-time/self-employed/unemployed/retired/students*	277 (99.28)	541 (88.11)	0.05 (0.01–22)	<0.001*
**Cancer related characteristics**				
Type of cancer†				
*Lung cancer*	66 (23.66)	160 (26.06)	1.14 (0.82–1.58)	0.44
*Gastric cancer*	56 (20.07)	51 (8.31)	0.36 (0.24–0.54)	<0.001*
*Liver cancer*	11 (3.94)	15 (2.44)	0.61 (0.28–1.35)	0.22
*Colorectal cancer*	38 (13.62)	113 (18.40)	1.43 (0.96–2.13)	0.08
*Ovarian cancer*	19 (6.83)	60 (9.77)	1.48 (0.87–2.53)	0.15
*Other cancers*	100 (35.84)	262 (42.67)	1.33 (0.99–1.78)	0.054
Metastatic cancers				
*No*	267 (95.70)	568 (92.51)	1	
*Yes*	12 (4.30)	46 (7.49)	1.80 (0.94–3.46)	0.08
**Presence of other chronic diseases, yes (vs. no)**				
Diabetes mellitus	19 (6.81)	25 (4.07)	0.58 (0.31–1.07)	0.08
Hypertension and/or hyperlipidemia	47 (16.85)	62 (10.10)	0.55 (0.37–0.83)	<0.001*
Chronic cardiovascular diseases	19 (6.81)	9 (1.47)	0.20 (0.09–0.46)	<0.001*
Chronic respiratory diseases	3 (1.08)	2 (0.33)	0.30 (0.05–1.81)	0.19
Chronic liver and/or kidney diseases	6 (2.15)	5 (0.81)	0.37 (0.11–1.23)	0.11
Other chronic diseases	4 (1.43)	17 (2.77)	1.96 (0.65–5.87)	0.23

CI – confidence interval, OR – odds ratio

*P < 0.001.

†Multiple-selection question.

### The uptake of the first COVID-19 vaccine booster dose and individual-level characteristics related to COVID-19 vaccination

Our study showed that individuals aged over 65 years of age had a lower uptake of the first COVID-19 vaccine booster dose in comparison to those under 65 years (23.66 vs. 31.92%, odds ratio (OR) = 1.51; *P* < 0.05) (**Table 2**). However, there were no significant differences between the two age groups in terms of hesitancy to uptake the first and second COVID-19 vaccine booster doses (73.24 vs. 70.57%, *P* = 0.48, 65.95 vs. 64.50%, *P* = 0.67). Furthermore, there were no significant differences in individual-level variables concerning the first COVID-19 vaccine booster dose, such as positive attitudes, negative attitudes, perceived subjective norm, perceived behavioural control, vaccination fatigue, consideration about the veracity of COVID-19 specific information, current treatment for cancer, and type of other chronic diseases (*P* > 0.05).

**Table 2:** Descriptive statistics of the first COVID-19 booster dose uptake, vaccine hesitancy, individual-level variables (n = 893)

	> = 65 y	<65 y	OR (95% CI)	*P*-value
	**n (%)**	**n (%)**		
**Uptake of the COVID-19 vaccine booster dose (the third dose)**				
No	213 (76.34)	418 (68.08)	1	
Yes	66 (23.66)	196 (31.92)	1.51 (1.09–2.09)	0.01*
**Hesitancy to receive COVID-19 vaccine booster doses**				
Hesitancy to receive the first COVID-19 vaccine booster dose (the third dose)				
*No (likely/very likely)*	57 (26.76)	123 (29.43)	1	
*Yes (very unlikely/unlikely/neutral)*	156 (73.24)	295 (70.57)	0.88 (0.6–1.27)	0.48
Hesitancy to receive the second COVID-19 vaccine booster dose (the fourth dose)				
*No (likely/very likely)*	95 (34.05)	218 (35.50)	1	
*Yes (very unlikely/unlikely/neutral)*	184 (65.95)	396 (64.50)	0.94 (0.70–1.26)	0.67
**Individual-level variables**				
Positive attitudes toward COVID-19 vaccine booster dose, n (%) agree/strongly agree				
*Receiving a booster dose can maintain your antibody level and strengthen the protection against COVID-19*	147 (52.69)	331 (53.91)	1.05 (0.79–1.39)	0.73
*A booster dose is highly effective in protecting you from COVID-19 variants of concern (e.g. Omicron)*	114 (40.86)	282 (45.93)	1.23 (092–1.64)	0.16
*A booster dose is highly effective in preventing severe consequences of COVID-19*	134 (48.03)	297 (48.37)	1.01 (0.76–1.35)	0.92
Negative attitudes toward COVID-19 vaccine booster dose, n (%) agree/strongly agree				
*The protection offered by COVID-19 vaccine booster dose is weaker among people with cancers*	111 (39.78)	245 (39.90)	1.00 (0.75–1.34)	0.97
*Cancer therapy would reduce the protection of COVID-19 vaccine booster dose*	98 (35.13)	211 (34.36)	0.97 (0.72–1.30)	0.82
*The side effects of the COVID-19 vaccine booster dose are more severe among people with cancers*	126 (45.16)	260 (42.35)	0.89 (0.67–1.19)	0.43
*The duration of protection of COVID-19 vaccine booster dose is shorter among people with cancers*	95 (34.05)	213 (34.69)	1.03 (0.76–1.39)	0.85
*COVID-19 vaccine booster dose would negatively affect the control of cancers*	118 (42.29)	262 (42.67)	1.02 (0.76–1.35)	0.92
Perceived subjective norm related to COVID-19 vaccine booster dose, n (%), agree/strongly agree.				
*Doctors would support you to uptake a booster dose*	57 (20.43)	138 (22.48)	1.13 (0.80–1.60)	0.49
*Family members would support you to uptake a booster dose*	69 (24.73)	174 (28.34)	1.20 (0.87–1.66)	0.26
Perceived behavioural control to receive a COVID-19 vaccine booster dose, n (%), agree/strongly agree				
*Receiving a COVID-19 vaccine booster dose is easy for you if you want to*	89 (31.90)	227 (36.79)	1.25 (0.93–1.69)	0.14
**Vaccination fatigue, n (%) agree/strongly agree**				
You are tired of receiving COVID-19 vaccinations over and over again	55 (19.71)	95 (15.47)	0.75 (0.52–1.08)	0.12
**Frequency of thoughtful consideration about the veracity of COVID-19-specific information**				
No (almost never/seldom)	130 (46.59)	260 (42.35)	1	
Yes (sometimes/always)	149 (53.41)	354 (57.65)	1.19 (0.89–1.58)	0.24

CI – confidence interval, COVID-19 – coronavirus disease 2019, OR – odds ratio

*P < 0.05.

### Factors associated with the uptake of the first COVID-19 vaccine booster dose

Univariate analysis revealed a statistically significant association between the Guangdong study site and the elderly cancer population regarding the uptake of the first COVID-19 booster dose (*P* < 0.05). However, no significant differences were found based on gender, ethnicity, education, relationship, and employment status (*P* > 0.05). For those under 65, individuals with higher education had a higher booster dose uptake (OR = 1.68; 95% confidence interval (CI) = 1.08–2.62, *P* < 0.05), while those without full-time jobs had a lower uptake (OR = 0.56; 95% CI = 0.34–0.92, *P* < 0.05) (Table S1 in the **Online Supplementary Document**).

The multivariable logistic regression was used to adjust for significant background variables (*P* < 0.05) in predicting the uptake of the first COVID-19 vaccine booster dose. For participants 65 and older, negative perceptions about COVID-19 vaccination, such as concerns about severe side effects (AOR = 0.55; 95% CI = 0.30–0.98), deterred booster dose uptake. Meanwhile, having one chronic disease reduced uptake (AOR = 2.39; 95% CI = 1.22–4.69). Doctor and family support (AOR = 3.50; 95% CI = 1.78–6.89) and perceived behavioural control (AOR = 2.47; 95% CI = 1.35–4.52) significantly promoted booster uptake (AOR = 2.13; 95% CI = 1.20–3.77). For those under 65, the support of doctors and family (AOR = 2.47; 95% CI = 1.64–3.72), perceived behavioural control (AOR = 2.24; 95% CI = 1.54–3.27), and frequent consideration of the veracity of COVID-19 information (AOR = 1.96; 95% CI = 1.37–2.80) significantly increased booster dose uptake (AOR = 1.70; 95% CI = 1.15–2.51) (Table S2 in the **Online Supplementary Document**).

### Differences in adverse reactions of uptake of the first COVID-19 vaccine booster dose in two age groups

Of the 262 cancer patients who received the first COVID-19 vaccine booster dose, 255 (97.33%) reported experiencing vaccine-related adverse reactions (response rate). The incidence of having at least one adverse reaction was similar between patients aged 65 and above, and patients under 65 reported similar (7.81 vs. 14.14%, *P* = 0.19). Additionally, the incidence of having each specific adverse reaction (pain, itching, etc.) was also similar between the two study groups (*P* > 0.05) (**Table 3**). The distribution of the brand of the first COVID-19 vaccine booster dose as shown in Table S3 in the **Online Supplementary Document**.

**Table 3:** Distribution of the first COVID-19 vaccine booster dose adverse reactions among the participants according to the two age groups (n = 255)

Outcome	> = 65 y	<65 y	*P*-value
	**n (%)**	**n (%)**	
Pain, itching, and swelling at the vaccination site	4 (6.25)	22 (11.52)	0.22
Fatigue	1 (1.56)	4 (2.09)	1.00
Vomiting and anorexia	0 (0.00)	4 (2.09)	0.58
Muscle joint headaches	1 (1.56)	3 (1.57)	1.00
Coughs	0 (0.00)	1 (0.52)	1.00
Fever	0 (0.00)	0 (0.00)	N.A.
Other adverse reactions	1 (1.56)	1 (0.52)	0.44
At least one adverse reaction	5 (7.81)	27 (14.14)	0.19

N.A. – not applicable

## DISCUSSION

This study found that only less than one fourth of cancer patient participants aged 65 years and above uptake the first COVID-19 booster vaccine, which is lower than that of adult cancer patients younger than 65 years and the general population in China. This is a very dangerous signal for the prevention and control of the COVID-19 epidemic in China, indicating that once the COVID-19 epidemic breaks out, cancer patients over 65 years old will have a very high risk of infection and mortality. Factors affecting individual-level variables among the aged 65 and above cancer patients group whether to uptake the COVID-19 booster dose were mainly several negative attitudes toward COVID-19 vaccine booster dose, perceived subjective norm, perceived behavioural control, and other types of chronic diseases. This study fills a gap in knowledge on the uptake of COVID-19 booster doses among cancer patients aged 65 and above and provides first-hand evidence for understanding the high mortality rate among elderly individuals in China following the release of COVID-19 prevention and control policies. The findings also provide direct information for conducting targeted interventions to increase the coverage of COVID-19 vaccine booster doses in elderly cancer patients.

In this study, we found that the vaccination rate for the first COVID-19 vaccine booster doses among cancer patients aged 65 and above was 23.66%, which is lower than the vaccination rate of elderly individuals aged 60 and above in China (53.2%) [23], as well as the vaccination rates of elderly individuals aged 65 and above in Japan (88.92% as of June 2022) [31]. In addition, we found that the rate for the first COVID-19 vaccine booster doses among cancer patients aged 65 and above was lower than in those younger than 65 years (31.92%). One plausible explanation for the lower vaccination rates among elderly cancer patients seems to be information problems consisting of questions about safety, inadequate information, conspiracy theories, and misinformation [32,33]. Therefore, government departments and mainstream media should disseminate reliable information from official sources to avoid misinformation subscriptions among older cancer populations. Some studies have shown that personal mobile communication, such as text messages that highlight fundamental information about COVID-19 vaccines, social welfare, and the benefits of group immunisation, could also improve vaccine acceptance [34–36].

Moreover, our study suggests that the presence of co-existing chronic diseases can influence the decision of elderly cancer patients aged 65 and above to uptake the first COVID-19 vaccine booster dose. Notably, patients with one chronic disease are more likely to uptake the booster dose compared to those without other chronic diseases. This may be attributed to the implementation of China's Basic Public Health Services, which include health management of the elderly, chronic disease management, health education, and vaccination. Community health centers have established health records for individuals aged 65 and above, provided proactive and continuous services for those with chronic diseases, and provide regular health and related vaccination publicity for this key population to promote vaccinations [37]. Although no significant correlation was found in the population under 65 years of age with co-existing chronic diseases., the government should develop specific educational interventions for cancer patients with co-existing chronic diseases, who are a high-risk group; the interventions should aim to increase their awareness, and knowledge of COVID-19 booster dose and help them make vaccination decisions.

We have found that negative attitudes toward COVID-19 vaccine booster dose, including the potential for more severe side effects, shorter duration of protection, and negative impact on cancer control, are significant barriers to vaccination among elderly cancer patients aged 65 and above. However, a third dose of booster vaccine was safe for vulnerable people in an Italian study, with no unexpected adverse reactions [38]. Currently, no evidence shows that the COVID-19 vaccine can cause cancer recurrence [39]. Instead, additional COVID-19 vaccines can improve the immunity of cancer patients significantly [40,41]. Thus, the benefits of COVID-19 vaccination significantly outweigh the risk for all cancer patients compared to the general population. The health government should leverage scientific vaccine projects to establish a more transparent, stable, and reasonable vaccination process. This can help address misunderstandings surrounding vaccination risks and dispel incorrect information through proper science popularisation and relevant instructions, ultimately strengthening the confidence of cancer patients aged 65 years and older in COVID-19 vaccination efforts.

Our study highlights the crucial role of family members and doctors' involvement in promoting the uptake of the first COVID-19 booster dose among older cancer patients. Doctors are responsible for providing complete and transparent cancer patients with the first COVID-19 booster dose. Due to the complexity of cancer occurrence and progression, physicians and clinicians managing elderly cancer patients should evaluate them based on cancer type, treatment stage, and treatment plan and recommend the uptake of the COVID-19 vaccine booster at an appropriate time [42].

### Study limitations

Several limitations of this study should be acknowledged. First, as a cross-sectional study, it cannot establish a causal relationship, and therefore, longitudinal studies are necessary to verify causality. Second, the rapidly changing policies and guidelines related to COVID-19 vaccination may affect the willingness of elderly cancer patients to accept the vaccine over time. Finally, the survey was conducted among cancer patients in only four urban hospitals in China, and the results may not fully represent the current vaccination willingness of all cancer patients aged 65 and above throughout China. Furthermore, the higher proportion of males and Han ethnicity in the sample might affect the research sample's representativeness. Therefore, it is essential to exercise caution when extrapolating the research findings. Despite the limitations mentioned above, this study is the first to reveal the factors influencing the vaccination behaviour of cancer patients aged 65 and older regarding the first COVID-19 booster dose, which could provide a theory for subsequent immunisation strategies for elderly cancer populations.

## CONCLUSIONS

The low uptake of the first COVID-19 booster dose among cancer patients aged 65 and above in China is a cause for concern that health departments should not overlook. To enhance vaccination rates in this population, health care departments and personnel need to grasp the crucial factors influencing their vaccination decisions from the patients' perspective. Specifically, the need to develop and execute targeted educational and promotional campaigns to enhance cancer patients' understanding and awareness of the COVID-19 vaccine. Moreover, during patient interactions, health care providers should offer information about the COVID-19 vaccine, including its efficacy, safety, and potential health benefits. Our research underlines the importance of considering these decisive factors in policy-making to better meet the needs of elderly cancer patients and thereby increase their vaccination rates. These findings can guide the formulation of more targeted vaccination promotion strategies, particularly for high-risk groups such as elderly cancer patients.

## Additional material


Online Supplementary Document
