# Supplementary material for: Low uptake of COVID-19 booster doses among elderly cancer patients in China: A multicentre cross-sectional study
Source: J Glob Health. 2024 Feb 2;14:05010. doi: 10.7189/jogh.14.05010 (PMC10835334; doi:10.7189/jogh.14.05010)
Supplement: Online Supplementary Document [file jogh-14-05010-s001.pdf]

Table S1 Univariate logistic regression of associations between background characteristics and uptake the first COVID-19 vaccine booster dose

Characteristics	Factors correlated with uptake the COVID-19 vaccine booster dose in the aged 65 and above			Factors correlated with uptake the COVID-19 vaccine booster dose in the <65 years		
	Prevalence of vaccine booster, n/N (%)	OR (95% CI)	P values	Prevalence of vaccine booster, n/N (%)	OR (95% CI)	P values
Gender						
Male	44/176(25.0)	1		104/310(33.55)		
Female	22/103(21.4)	0.81(0.46, 1.46)	0.49	92/304(30.26)	0.86(0.61, 1.21)	0.38
Study site						
Xinjiang	29/91(31.87)	1		93/190(48.95)	1	
Inner Mongolia	10/73(13.70)	0.34(0.15, 0.76)	0.01[*]	28/134(20.90)	0.28(0.17, 0.46)	<0.001[**]
Shanxi	20/79(25.32)	0.72(0.37, 1.42)	0.35	39/126(30.95)	0.47(0.29, 0.75)	<0.001[**]
Guangdong	7/36(19.44)	0.52(0.20, 1.32)	0.17	36/164(21.95)	0.29(0.18, 0.47)	<0.001[**]
Ethnicity						
Other ethnic minorities	9/38(23.68)	1		42/112(37.50)	1	
Han majority	57/241(23.65)	1.00(0.45, 2.23)	1.00	154/502(30.68)	0.74(0.48, 1.13)	0.16
Education level						0.64
Junior high or below	46/208(22.12)	1		113/391(28.90)	1	
Senior high or equivalent	15/49(30.61)	1.55(0.78, 3.10)	0.21	40/117(34.19)	1.28(0.82, 1.98)	0.27
College and above	5/22(22.73)	1.04(0.36, 2.96)	0.95	43/106(40.57)	1.68(1.08, 2.62)	0.02[*]
Relationship status						
Single/divorced/widowed	5/28(17.86)	1		13/49(26.53)	1	
Married	61/251(24.30)	1.48(0.54, 4.05)	0.45	183/565(32.39)	1.33(0.69, 2.56）	0.40
Employment status						
Full-time	0/2(0.00)	1		32/73(43.84)	1	
Part-time/self-employed/ unemployed/retired/students	66/277(23.83)	N.A.	N.A.	164/541（30.31）	0.56(0.34, 0.92)	0.02[*]
[†]Type of cancer						
Lung cancer	15/66(22.73)	0.93(0.48, 1.80)	0.84	47/160(29.38)	0.85(0.57, 1.26)	0.42

Gastric cancer	15/56(26.79)	1.23(0.63, 2.41)	0.54	20/51(39.22)	1.42(0.79, 2.56)	0.25
Liver cancer	5/11(45.45)	2.83(0.83, 9.59)	0.10	5/15(33.33)	1.07(0.36, 3.17)	0.91
Colorectal cancer	8/38(21.05)	0.84(0.37, 1.94)	0.68	30/113(26.55)	0.73(0.46, 1.15)	0.18
Ovarian cancer	3/19(15.79)	0.59(0.17, 2.08)	0.41	21/60(35.00)	1.17(0.67, 2.04)	0.59
Other cancers	23/100(23.00)	0.94(0.53, 1.68)	0.85	88/262(33.59)	1.14(0.81, 1.61)	0.45
Metastatic cancers						
No	63/267(23.60)	1		182(29.64)	1	
Yes	3/12(25.00)	1.08(0.28,4.11)	0.91	14(2.28)	0.93(0.48, 1.78)	0.82

Note：

* *P*<0.05;

** *P*<0.001;

OR: Crude Odds Ratios;

CI: Confidence Interval;

† Multiple-selection question.

Table S2 Multivariable logistic regression of factors correlated with uptake the first COVID-19 vaccine booster dose among multi-center cancer participants

Characteristics	Factors correlated with uptake the COVID-19 vaccine booster dose in the aged 65 and above					Factors correlated with uptake the COVID-19 vaccine booster dose in the <65 years				
	Prevalence of vaccine booster, n/N (%)	OR (95% CI)	P values	AOR (95% CI)	P values	Prevalence of vaccine booster, n/N (%)	OR (95% CI)	P values	AOR (95% CI)	P values
Individual-level variables										
Views on COVID-19 vaccine booster dose										
Positive attitudes toward COVID-19 vaccine booster dose, n/N () agree/strongly agree										
Receiving a booster dose can maintain your antibody level and strengthen the protection against COVID-19	35/147 (23.81)	1.02 (0.59,1.77)	0.95	0.94 (0.53, 1.65)	0.82	31/132 (23.48)	0.95 (0.68, 1.34)	0.77	0.75 (0.52, 1.08)	0.12
A booster dose is highly effective in protecting you	33/114 (28.95)	1.63 (0.93,2.84)	0.09	1.55 (0.88, 2.72)	0.13	98/282 (34.75)	1.27 (0.91, 1.79)	0.17	1.00 (0.69, 1.43)	0.98

from COVID-19 variants of concern (e.g., Omicron)										
A booster dose is highly effective in preventing severe consequences of COVID-19	30/134 (22.39)	0.87 (0.50,1.52)	0.63	0.83 (0.47, 1.45)	0.50	105/297 (35.35)	1.36 (0.97, 1.91)	0.08	1.08 (0.75, 1.55)	0.67
Negative attitude toward COVID-19 vaccine booster dose, n/N () agree/strongly agree										
The protect;ion offered by COVID-19 vaccine booster dose is weaker among people with cancers	21/111 (18.92)	0.64 (0.36, 1.14)	0.13	0.66 (0.37, 1.20)	0.17	76/245 (31.02)	0.93 (0.66, 1.32)	0.70	1.00 (0.70, 1.43)	0.99
Cancer therapy would reduce the protection of COVID-19 vaccine booster dose	19/98 (19.39)	0.69 (0.38, 1.25)	0.22	0.69 (0.38, 1.27)	0.23	64/211 (30.33)	0.89 (0.62, 1.28)	0.54	1.02 (0.70, 1.48)	0.92
The side effects of COVID-19 vaccine booster dose are more	22/126 (17.36)	0.52 (0.29, 0.93)	0.03*	0.55 (0.30, 0.98) .	0.04*	71/260 (27.31)	0.69 (0.49, 0.98)	0.04*	0.79 (0.55, 1.13)	0.20

severe among people with cancers										
The duration of protection of COVID-19 vaccine booster dose is shorter among people with cancers	15/95 (15.79)	0.49 (0.26, 0.93)	0.03*	0.49 (0.26, 0.94)	0.03*	67/213 (31.46)	0.97 (0.68, 1.38)	0.86	1.18 (0.81, 1.72)	0.38
COVID-19 vaccine booster dose would negatively affect the control of cancers	19/118 (16.10)	0.47 (0.26, 0.85)	0.01*	0.48 (0.26, 0.87)	0.02*	72/262 (27.48)	0.70 (0.49, 0.99)	0.04*	0.79 (0.55, 1.14)	0.21
Perceived subjective norm, n/N () agree/strongly agree										
Doctors would support you to uptake a booster dose	25/57 (43.86)	3.45 (1.85, 6.43)	<0.001	3.50 (1.78, 6.89)	<0.001 **	72/138 (52.17)	3.10 (2.09, 4.58)	<0.001 **	2.47 (1.64, 3.72)	<0.001 **
Family member would support you to uptake a booster dose	26/69 (37.68)	2.57 (1.42, 4.67)	0.00*	2.47 (1.35, 4.52)	0.03*	82/174 (47.13)	2.55 (1.77, 3.68)	<0.001 **	2.24 (1.54, 3.27)	<0.001 **
Perceived behavioral control ,n/N () agree/strongly agree										
Receiving a COVID-19	30/89 (33.71)	2.18 (1.23, 3.85)	0.01*	2.13 (1.20, 3.77)	0.01*	97/227 (42.73)	2.17 (1.53, 3.07)	<0.001 **	1.96 (1.37, 2.80)	<0.001 **

vaccine booster dose is easy for you if you want to										
Vaccination fatigue (tired of receiving COVID-19 vaccination over and over again)	11/55 (20.00)	0.74 (0.59,0.93)	0.01*	0.74 (0.36, 1.54)	0.42	35/95 (36.84)	1.30 (0.82, 2.05)	0.26	1.17 (0.73, 1.87)	0.51
Frequency of thoughtful consideration about veracity of COVID-19-specific information (Sometimes/ Always)	38/149 (25.50)	1.25 (0.71, 2.18)	0.44	1.12 (0.62, 2.03)	0.71	138/354 (39.00)	2.23 (1.55, 3.19)	0.00*	1.70 (1.15, 2.51）	0.01*
Current treatment for cancer										
Not yet started treatment	4/9 (44.44)	1		1		8/18 (44.44)	1		1	
Chemotherapy only	41/186 (22.04)	0.35 (0.09,1.38)	0.13	0.36 (0.09,1.40)	0.14	110/430 (25.58)	0.43 (0.17, 1.12)	0.08	0.55 (0.21, 1.45)	0.23
Radiotherapy only	14/37 (37.84)	0.76 (0.17, 3.32)	0.72	0.66 (0.14, 3.03)	0.59	47/78 (60.26)	1.90 (0.67, 5.33)	0.23	1.71 (0.59, 4.94)	0.32
Immunotherapy only	0/8 (0.00)	N.A.	N.A.	N.A.	N.A.	3/15 (20.00)	0.31 (0.07, 1.50)	0.15	0.34 (0.07, 1.64)	0.18
Chemotherapy and radiotherapy	1/13 (7.69)	0.10 (0.01,1.18)	0.07	0.09 (0.01,1.07)	0.06	16/39 (41.03)	0.87 (0.28, 2.69)	0.81	0.76 (0.24, 2.41)	0.65

Immunotherapy and chemotherapy /radiotherapy	3/15 (20.00)	0.31 (0.05,1.94)	0.21	0.31 (0.05,1.93)	0.21	4/14 (28.57)	0.50 (0.11, 2.21)	0.36	0.62 (0.14, 2.82)	0.54
Treatment completed	3/11 (27.27)	0.47 (0.07,3.04)	0.43	0.47 (0.07,3.05)	0.43	8/20 (40.00)	0.83 (0.23, 3.03)	0.78	1.13 (0.30, 4.23)	0.85
Type of other chronic diseases										
0	47/207 (22.71)	1		1		159/519 (30.64)	1			
1	19/47 (40.43)	2.31 (1.19, 4.50)	0.01*	2.39 (1.22, 4.69)	0.01*	29/73 (39.73)	1.49 (0.90, 2.47)	0.12	1.50 (0.89, 2.52)	0.13
>=2	0/25 (0.00)	N.A.	N.A.	N.A.	N.A.	8/22 (36.36)	1.29 (0.53, 3.15)	0.57	1.18 (0.47, 2.98)	0.72

Note：
* P<0.05;
** P<0.001;
CI: Confidence Interval;
OR: Crude Odds Ratios;

AOR: Adjusted Odds Ratios, odds ratios adjusted for significant background characteristics listed in Table S1.

Table S3 Distribution of the brand of the first COVID-19 vaccine booster dose among participants across two age groups (N = 262)

Brand	>=65 years	<65 years
	N(%)	N(%)
CoronaVac	30(45.45)	90(45.92）
BBIBP-CorV	11(16.67)	27(13.78)
Shenzhen Kangtai	1(1.52)	3(1.53)
Anhui Zhifei	3(4.55)	18(9.18)

Ad5-nCoV	0(0.00)	2(1.02)
Others	21(31.82)	56(28.57)

STROBE Statement—Checklist of items that should be included in reports of ***cross-sectional studies***

	Item No	Recommendation
**Title and abstract**	1<br>Page 2	(*a*) Indicate the study's design with a commonly used term in the title or the abstract
		(*b*) Provide in the abstract an informative and balanced summary of what was done and what was found
**Introduction**		
Background/rationale	2<br>Page3~4	Explain the scientific background and rationale for the investigation being reported
Objectives	3<br>Page 4	State specific objectives, including any prespecified hypotheses
**Methods**		
Study design	4<br>Page 4~5	Present key elements of study design early in the paper
Setting	5<br>Page 4~5	Describe the setting, locations, and relevant dates, including periods of recruitment, exposure, follow-up, and data collection
Participants	6<br>Page 5	(*a*) Give the eligibility criteria, and the sources and methods of selection of participants
Variables	7<br>Page 5	Clearly define all outcomes, exposures, predictors, potential confounders, and effect modifiers. Give diagnostic criteria, if applicable

Data sources/ measurement	8* Page 5-6	For each variable of interest, give sources of data and details of methods of assessment (measurement). Describe comparability of assessment methods if there is more than one group
Bias	9	Describe any efforts to address potential sources of bias
Study size	10 Page 6	Explain how the study size was arrived at
Quantitative variables	11 Page 5-6	Explain how quantitative variables were handled in the analyses. If applicable, describe which groupings were chosen and why
Statistical methods	12 Page 6-7	(*a*) Describe all statistical methods, including those used to control for confounding
		(*b*) Describe any methods used to examine subgroups and interactions
		(*c*) Explain how missing data were addressed
		(*d*) If applicable, describe analytical methods taking account of sampling strategy
		(*e*) Describe any sensitivity analyses
**Results**		
Participants	13* Page 7	(a) Report numbers of individuals at each stage of study—eg numbers potentially eligible, examined for eligibility, confirmed eligible, included in the study, completing follow-up, and analysed
		(b) Give reasons for non-participation at each stage
		(c) Consider use of a flow diagram
Descriptive data	14* Page 7-8	(a) Give characteristics of study participants (eg demographic, clinical, social) and information on exposures and potential confounders
		(b) Indicate number of participants with missing data for each variable of interest

Outcome data	15*	Report numbers of outcome events or summary measures
Main results	16<br>Page 7-11	(*a*) Give unadjusted estimates and, if applicable, confounder-adjusted estimates and their precision (eg, 95% confidence interval). Make clear which confounders were adjusted for and why they were included
		(*b*) Report category boundaries when continuous variables were categorized
		(*c*) If relevant, consider translating estimates of relative risk into absolute risk for a meaningful time period
Other analyses	17	Report other analyses done—eg analyses of subgroups and interactions, and sensitivity analyses
**Discussion**		
Key results	18<br>Page 11-13	Summarise key results with reference to study objectives
Limitations	19<br>Page 13	Discuss limitations of the study, taking into account sources of potential bias or imprecision. Discuss both direction and magnitude of any potential bias
Interpretation	20<br>Page 13	Give a cautious overall interpretation of results considering objectives, limitations, multiplicity of analyses, results from similar studies, and other relevant evidence
Generalisability	21	Discuss the generalisability (external validity) of the study results
**Other information**		
Funding	22<br>Page 15	Give the source of funding and the role of the funders for the present study and, if applicable, for the original study on which the present article is based

*Give information separately for exposed and unexposed groups.

Note: An Explanation and Elaboration article discusses each checklist item and gives methodological background and published examples of transparent reporting. The STROBE checklist is best used in conjunction with this article (freely available on the Web sites of PLoS Medicine at http://www.plosmedicine.org/, Annals of Internal Medicine at http://www.annals.org/, and Epidemiology at http://www.epidem.com/). Information on the STROBE Initiative is available at www.strobe-statement.org.
